# Optimizing clavicle hook plate fixation through biomechanical analysis of pre-bent plate condition and screw configurations

**DOI:** 10.3389/fmed.2026.1752369

**Published:** 2026-03-17

**Authors:** Pengfei Nie, XueYi He, Zhengchao Zhang, Pinhua Chen, Ruoli Wang, Qi Fang, Jian Guo, Wubing He

**Affiliations:** 1 Department of Emergency Trauma Surgery, Shengli Clinical Medical College of Fujian Medical University, Fuzhou, Fujian, China; 2 Fuzhou University Affiliated Provincial Hospital, Fuzhou, Fujian, China; 3 Department of Orthopaedic, Beilun District People’s Hospital, Ningbo, Zhejiang, China; 4 Department of Emergency Trauma Surgery, Fujian Provincial Hospital, Fuzhou, Fujian, China; 5 Fujian Trauma Medicine Center, Fuzhou, Fujian, China; 6 Fujian Key Laboratory of Emergency Medicine, Fuzhou, Fujian, China

**Keywords:** biomechanical stability, clavicle hook plate, empty screw hole, fixation strength, plate bending, screw type

## Abstract

**Objective:**

Clavicle hook plates are widely used for internal fixation of clavicle fractures, yet little biomechanical evidence exists to guide optimal plate contouring, screw selection, and screw-hole management. This study aimed to systematically evaluate the biomechanical effects of plate bending configuration, screw type, empty hole location, and invalid hole placement on clavicle hook plate fixation strength to optimize surgical strategies for clavicle fracture fixation.

**Methods:**

This was a controlled bench-top biomechanical study. A series of biomechanical tests were conducted using an electronic universal testing machine. Clavicle hook plates were fixed onto synthetic clavicle models under different experimental conditions: (1) plate bending (forward bend, no bend, reverse bend), (2) screw type (common screws vs. locked screws), (3) empty screw hole location (distal vs. proximal), and (4) invalid hole placement (under the plate vs. beyond the plate). Axial force was applied to the distal hook until fracture occurred, and the maximum fracture force was recorded. One-way ANOVA with *post-hoc* Bonferroni correction was used for statistical analysis (*p* < 0.01 considered significant).

**Results:**

Plate bending significantly influenced fixation strength, with the forward bend group exhibiting the highest fracture force (202.75 N), significantly greater than the no bend and reverse bend groups (*p* < 0.01). Common screws provided greater mechanical stability than locked screws, with significantly higher fracture force (204.08 N vs. 145.76 N, *p* < 0.0001). Distal empty screw holes significantly reduced fixation strength (135.38 N) compared to proximal empty holes (160.3 N, *p* < 0.0001). Invalid holes beyond the plate weakened structural integrity more than holes under the plate (144.75 N vs. 169.27 N, *p* < 0.0001).

**Conclusion:**

The study demonstrates that forward bending of the plate, the use of common screws, and avoiding distal empty screw holes or invalid holes beyond the plate significantly improve fixation strength in clavicle hook plate fixation. These findings provide critical biomechanical insights to enhance surgical decision-making and reduce the risk of implant failure. Future research should focus on clinical validation, multi-axial loading analysis, and long-term fatigue testing to further refine fixation techniques for optimal patient outcomes.

## Introduction

Clavicle fractures, particularly those involving the distal clavicle, are common orthopedic injuries that often require surgical fixation to ensure proper healing and functional recovery (1). The use of clavicle hook plates has been widely adopted for internal fixation, particularly for complex fractures near the acromioclavicular joint. These plates provide structural stability by anchoring the distal clavicle while allowing for early mobilization (2, 3).

However, complications such as implant failure, malunion, nonunion, and discomfort due to implant prominence remain significant challenges in clinical practice (4–6). Several factors influence the mechanical strength of clavicle hook plates, including plate bending configuration, screw type and placement, and the presence of empty or invalid screw holes in the clavicle (7–9). Although these factors have been studied individually, there is no consensus on the optimal biomechanical configuration to maximize fracture stability while minimizing implant failure. This gap in knowledge highlights the need for a systematic evaluation of these parameters under controlled experimental conditions.

Previous studies have investigated the influence of plate bending on mechanical stability. The rate of clavicular midshaft fracture after hook plate fixation was 6.0%. Some studies suggest that pre-bending the plate may improve bone-plate contact, thereby increasing fixation strength (10–12). However, the effect of different bending directions (forward or reverse) remains unclear, with conflicting results reported in the literature (13–16). In terms of screw selection, both common cortical screws and locked screws are used for clavicle fracture fixation. Common screws allow for compression at the fracture site, which may enhance stability, while locked screws provide fixed-angle support, potentially offering better resistance to angular deformation (17, 18). However, it is not well understood which screw type provides superior biomechanical performance in clavicle hook plate fixation. Additionally, empty screw holes in the plate can significantly impact construct stability. Studies on other orthopedic implants suggest that empty holes may act as stress concentrators, leading to early implant failure (19–21). However, the specific impact of empty holes in different locations on a clavicle plate has not been fully quantified. Lastly, the effect of drilled but unused (invalid) holes in the clavicle itself is a concern. Such holes may weaken bone integrity and reduce fracture resistance, yet there is limited biomechanical data on how their placement (under or beyond the plate) affects mechanical performance.

Despite these existing studies, no comprehensive biomechanical analysis has simultaneously evaluated the effects of plate bending, screw selection, empty holes, and invalid holes on the mechanical stability of clavicle hook plates. The lack of systematic comparative data makes it difficult for orthopedic surgeons to make evidence-based decisions when selecting implant configurations. This study aims to fill this critical research gap.

The primary objective of this study is to systematically evaluate the biomechanical effects of different plate bending configurations, screw types, empty hole locations, and invalid hole placements on clavicle hook plate fixation strength. Using a controlled mechanical testing setup, we will assess the impact of plate bending (forward, no bend, reverse) on fracture resistance, compare the biomechanical stability of common screws vs. locked screws in clavicle fixation, determine the effect of empty screw holes at distal and proximal locations on structural integrity, and investigate the influence of invalid holes under and beyond the plate on overall mechanical performance. By addressing these questions, this study aims to provide clinically relevant biomechanical insights that can inform optimal surgical techniques for clavicle fracture fixation. Our hypothesis is that forward-bend-plate, unlocked screw will decrease the incidence of clavicle fracture, but the distal-empty-hole plate will increase the incidence of clavicle fracture. Invalid hole covered by plate has enough strength to decrease the incidence of clavicle fracture, compare to normal clavicle.

## Methods

### Experimental design

**Figure 1:**
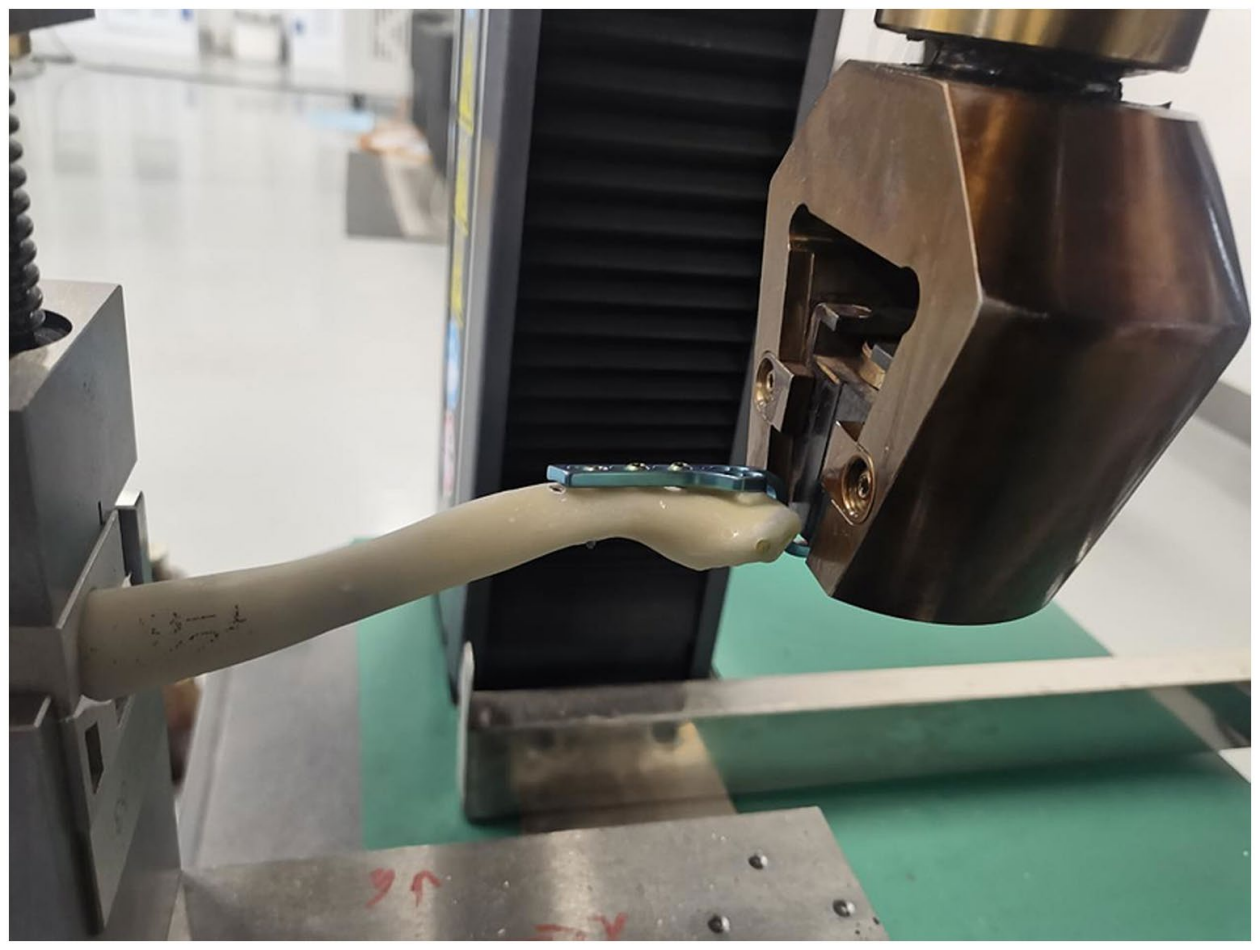
Electronic universal testing machine. Photograph of the mechanical testing apparatus used to evaluate construct stability. The clavicle hook plate was fixed in the distal synthetic clavicle specimens. The proximal clavicle was fixed in the machine. The compressive force was increasing on the distal hook until the clavicle was fracture, meanwhile the electronic machine will record the forces in the experiment.

This study investigated the mechanical stability of clavicle hook plates (Double Medical Technology Co., Ltd., Xiamen, China) under different bending conditions, screw types, and hole placements using an electronic universal testing machine (CMT4104GD, Shenzhen Sansi Testing Equipment Co., Ltd., China) (Figure 1). Synthetic clavicle bone models (Pacific Research Laboratories, Inc., Washington State, USA) were used to ensure uniformity across trials. The plates were fixed using cortical screws, and different experimental conditions were applied. The clavicle hook plate was secured at the distal clavicle while the proximal clavicle was fixed within the testing machine. A progressively increasing force was applied to the distal hook until fracture occurred. The force at fracture was recorded for all tested configurations. The experimental clavicle models include distal clavicle fractures (Neer type V) and acromioclavicular joint dislocations.

### Pre-bend plate conditions

**Figure 2:**
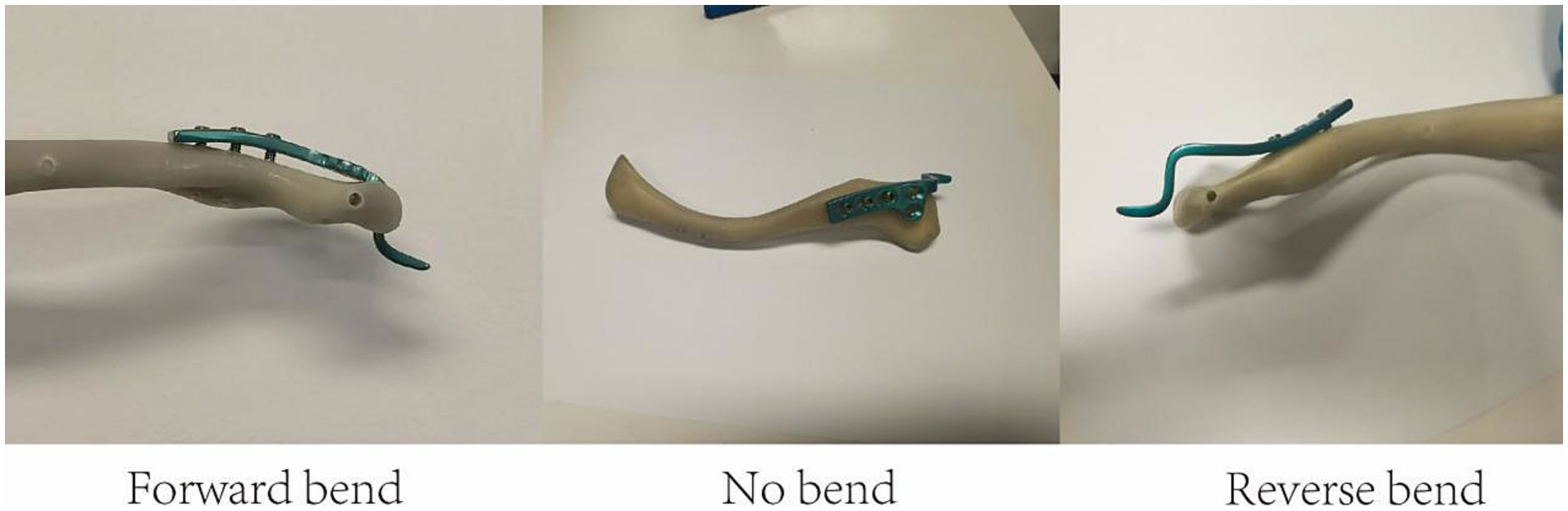
The bending plate experiments. Schematic illustration of the three plate bending configurations used in the study. The forward-bend group is that the middle of plate has a 1–2 cm gap to the surface of clavicle. The no-bend group is that the plate is not bend before fixed in the clavicle. The reverse-bend group is that the middle of plate is touch the clavicle and the two end of plate are raised up.

The experimental groups were designed to evaluate the biomechanical effects of plate bending configurations, screw types, and screw hole occupancy (Figure 2). Fifteen synthetic clavicle models were used in each group, and each specimen was used only once. In the forward-bend group, the central portion of the clavicle hook plate was elevated approximately 1–2 cm from the clavicle surface prior to fixation, simulating anterior curvature. The central angle of the plate arc is 0.54°, and the radius of curvature is 107 mm. In the no-bend group, the plate was applied in its native, unbent form without any pre-contouring. In the reverse-bend group, the center of the plate was in direct contact with the clavicle, while both ends were elevated, creating a posterior curvature. The central angle of the plate arc is 0.57°, and the radius of curvature is 101 mm. To assess the influence of screw type, two groups were established: the common screw group (Group A) used a conventional cortical screw at the proximal position, whereas the locked screw group (Group B) employed a locking screw in the same location. To evaluate the role of unfilled screw holes on construct stability, the distal empty hole group (Group C) had one unoccupied hole at the distal end of the plate, while the proximal empty hole group (Group D) featured an unfilled hole adjacent to the proximal screw.

### Invalid hole placement

**Figure 3:**
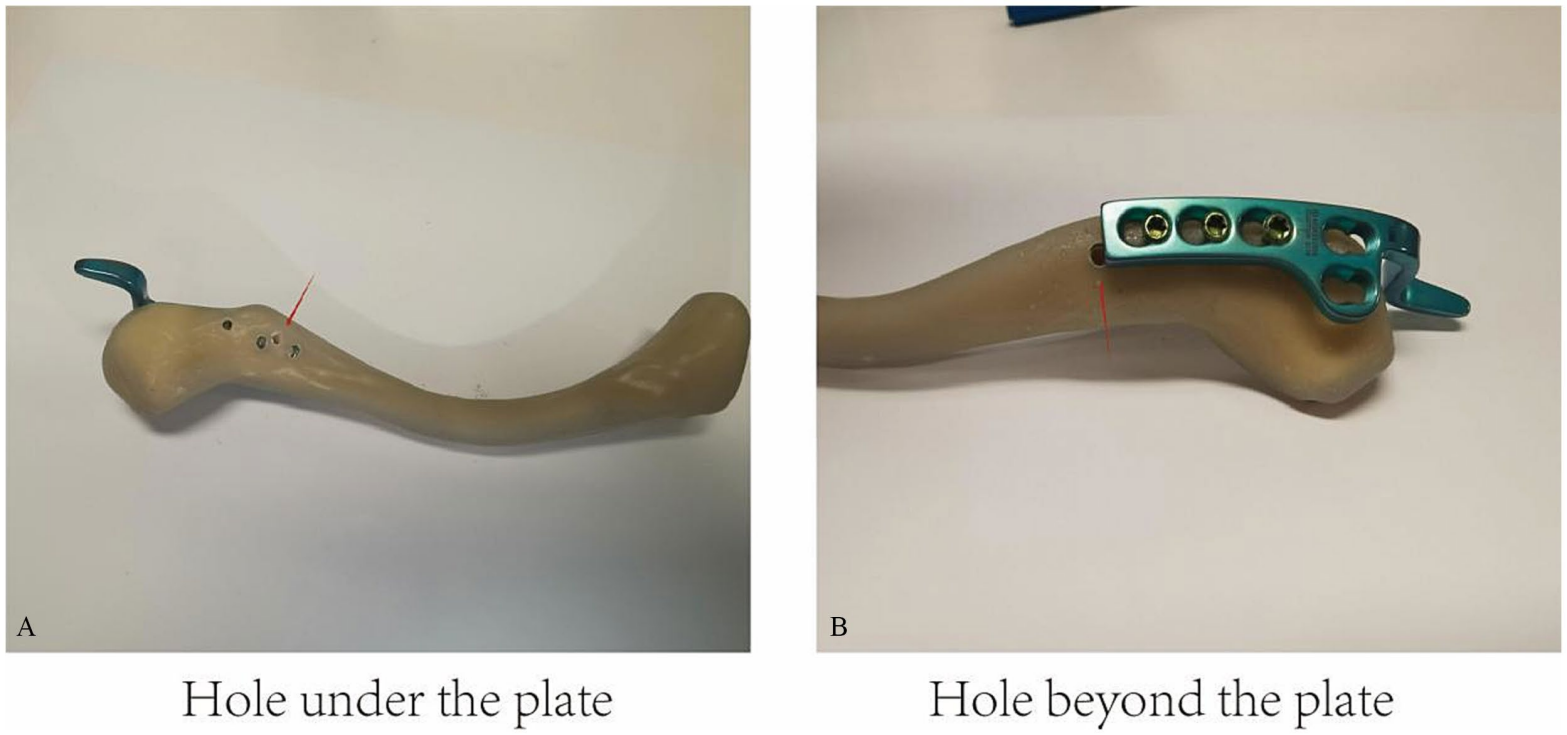
The influence of drilled an invaild hole in clavicle experiments. **(A)** Distal-empty-hole group (red arrow): a screw hole at the distal end of the plate was left unfilled. **(B)** Proximal-empty-hole group (red arrow): a screw hole near the proximal end of the plate was left unfilled.

To evaluate the influence of invalid hole positioning on the mechanical integrity of the construct, two configurations were established (Figure 3). Fifteen synthetic clavicle models were used in each group, and each specimen was used only once. In the “under-plate” condition, a cortical hole was drilled between the first and second proximal screw positions, located directly beneath the clavicle hook plate. In the “beyond-plate” condition, a similar hole was placed proximal to the plate, outside the area covered by the implant and corresponding to a theoretical proximal screw site if a longer plate were used. These configurations were designed to simulate unintended drilling during surgery and to assess the biomechanical consequences of misplaced or unused holes.

### Mechanical testing procedure

Each clavicle specimen was mounted in the electronic universal testing machine (Figure 1). A compressive force was applied at the distal hook of the clavicle plate at a constant rate until fracture occurred. The force at the point of fracture was automatically recorded.

### Mechanical testing of clavicle hook plates

The mechanical testing was conducted using an electronic universal testing machine (Figure 1). The clavicle hook plate was fixed in the distal clavicle while the proximal clavicle was secured in the machine. The applied force was progressively increased at the distal hook until clavicle fracture occurred, and the machine recorded the maximum fracture force.

The maximum fracture force (N) was measured for each specimen across all experimental conditions. Three independent sets of experiments were performed with bending conditions (Table 1), screw type and empty hole location (Table 2), and invalid hole placement (Table 3).

**Table 1:** The force data of bend plate groups.

Group	Maximum fracture force (N)														
Forward bend	202.75	200.98	203.76	201.88	197.94	197.93	203.81	205.42	200.53	200.64	207.75	207.87	205.85	203.43	199.11
No bend	138.76	144.78	144.57	134.9	138.7	144.57	143.86	140.37	142.91	144.82	142.74	145.95	135.5	136.7	141.65
Reverse bend	145.76	141.9	146.67	141.84	148.97	145.99	143.75	143.76	147.99	144.77	140.57	147.86	148.96	145.98	144.76

**Table 2:** The force data of screw type and location groups.

Group	Force (N)														
A (common screw)	202.33	205.78	205.54	204.86	203.42	204.17	205.48	199.35	198.17	205.79	200.88	206.45	204.44	199.16	204.08
B (lock screw)	145.76	148.56	145.67	150.36	148.9	144.85	142.75	146.63	149.74	142.83	149.06	143.07	141.14	149.16	140.27
C (distal empty hole)	138.76	143.26	135.38	133.57	140.38	133.68	135.47	137.86	135.26	139.65	140.36	143.05	137.4	143.08	135.38
D (proximal empty hole)	160.3	160.76	163.6	164.56	163.85	164.27	163.28	163.69	160.38	161.57	159.47	160.72	162.58	165.26	156.73

**Table 3:** The force data of invalid hole in the clavicle.

Group	Force (N)														
Hole under the plate	168.01	172.35	169.89	171.74	173.26	171.87	173.38	171.45	165.98	171.07	164.09	170.02	164.05	164.17	169.27
Hole beyond the plate	144.75	147.72	149.34	141.35	147.97	148.65	145.05	142.08	143.78	143.36	143.25	150.18	144.26	147.57	143.38

### Statistical analysis

**Figure 4:**
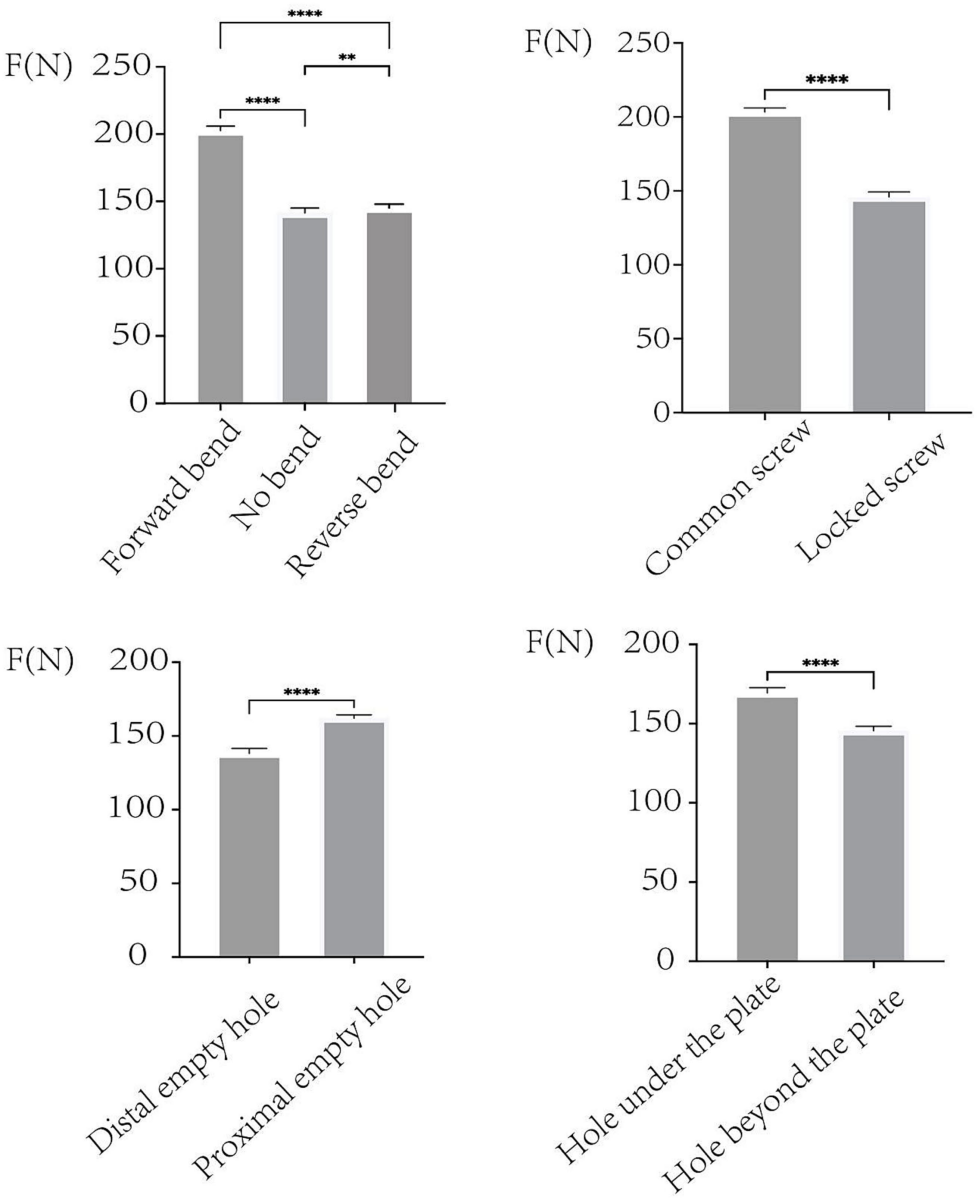
The maximum force of these groups were measured in the figures, when the clavicles were fractured. The force of forward-bend group is bigger than no-bend group and reverse-bend group. The force of common-screw group is bigger than locked screw. The force of distal-empty-hole group is smaller than the proximal-empty-hole group. The force of hole-under-the-plate group is bigger than the hole-beyond-the-plate group. Means *p* < 0.01, ****means *p* < 0.0001.

The analysis of the data was conducted using SPSS version 26.0 software, developed by IBM Corporation, based in Armonk, New York, USA. All data were analyzed using one-way ANOVA with *post-hoc* Bonferroni correction for multiple comparisons. The results were presented as mean ± standard deviation (SD). Statistical significance levels were set at *p* < 0.05. Statistical comparisons were visualized in Figure 4, where significant differences were observed between different bending conditions, screw types, and hole locations.

## Results

### Influence of pre-bent plate condition on maximum fracture force

The bending experiments investigated three different configurations: forward bend, no bend, and reverse bend (Figure 2). The results (Table 1) demonstrated that the forward bend group exhibited the highest fracture force (mean: 202.64 ± 3.20 N), significantly greater than both the no bend group (mean: 141.39 ± 3.67 N, *p* < 0.01) and the reverse bend group (mean: 145.30 ± 2.60 N, *p* < 0.01). Statistical analysis confirmed that the forward bend significantly improved structural stability, whereas the reverse bend and no bend groups had lower fracture resistance (Figure 4, top left).

### Influence of screw type on fracture resistance

**Figure 5:**
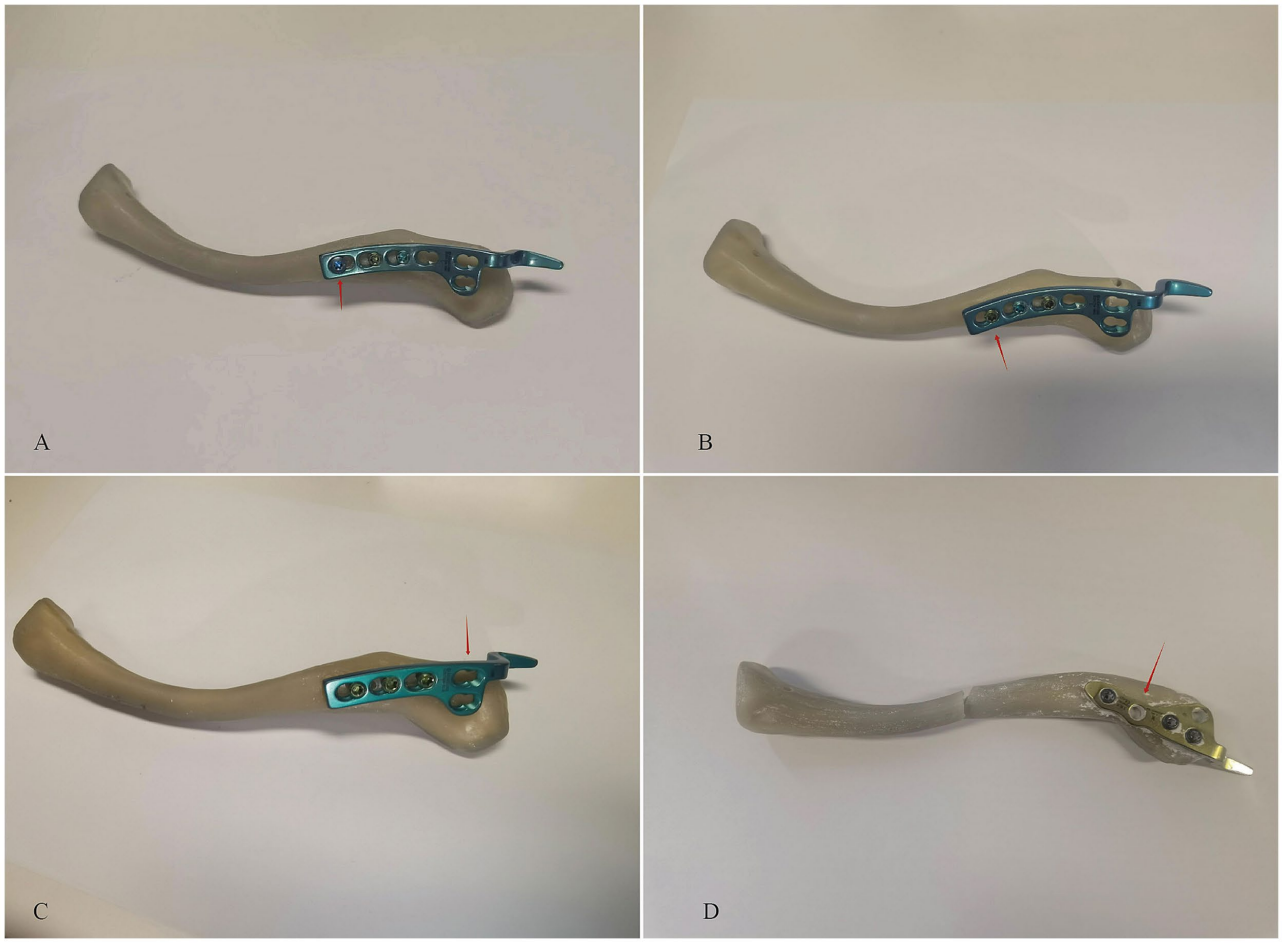
The screws type and location experiments. Comparison of screw types used at the proximal hole of the plate. **(A)** The proximal screw (red arrow) is common screw. **(B)** The proximal screw (red arrow) is lock screw. **(C)** The empty hole (red arrow) of the plate is located in the distal plate. **(D)** The empty hole (red arrow) of the plate is located near the proximal screw. The remaining holes of C, D plates were fixed by screws.

Comparing common screws and locked screws (Figures 5A,B), the results in Table 2 show that the common screw group exhibited a significantly higher fracture force (mean: 203.33 ± 2.71 N) compared to the locked screw group (mean: 145.92 ± 3.32 N, *p* < 0.0001). This suggests that common screws provide superior stability over locked screws in this experimental setup (Figure 4, top right).

### Influence of empty holes in the plate

The effect of empty holes at different locations was evaluated by comparing distal empty holes (Figure 5C) versus proximal empty holes (Figure 5D). Table 2 shows that the proximal empty hole group demonstrated significantly higher resistance (mean: 162.07 ± 2.33 N) compared to the distal empty hole group (mean: 138.17 ± 3.37 N, *p* < 0.0001). This indicates that the presence of an empty hole at the distal plate region compromises structural integrity more than a proximal empty hole (Figure 4, bottom left).

### Effect of invalid holes in the clavicle

The final set of experiments (Figure 3) examined the effect of drilled invalid holes in the clavicle, distinguishing between a hole located under the plate and a hole beyond the plate. The results (Table 3) indicate that the fracture force was significantly higher in the hole under the plate group (mean: 169.37 ± 3.34 N) compared to the hole beyond the plate group (mean: 145.51 ± 2.81 N, *p* < 0.0001). This suggests that the presence of an invalid hole beyond the plate weakens the structure more than a hole located directly beneath the plate (Figure 4, bottom right).

## Discussion

This study systematically evaluated the biomechanical effects of plate bending configuration, screw type, empty screw hole position, and the presence of invalid holes on the fixation strength of clavicle hook plates. The results demonstrated that plate bending significantly influences fracture resistance. The forward-bend group exhibited the highest fracture force (mean: 202.64 ± 3.20 N), while the no-bend and reverse-bend groups showed significantly lower fixation strength (*p* < 0.01). Additionally, common screws provided greater stability than locked screws. The fracture force in the common-screw group (mean: 203.33 ± 2.71 N) was significantly higher than in the locked-screw group (mean: 145.92 ± 3.32 N, *p* < 0.0001), suggesting that common screws may offer superior mechanical stability in clavicle hook plate fixation. The location of empty screw holes affected structural integrity. Plates with a distal empty screw hole had significantly lower fracture force (mean: 138.17 ± 3.37 N) compared to those with a proximal empty hole (mean: 162.07 ± 2.33 N, *p* < 0.0001). Furthermore, invalid holes in the clavicle weakened overall mechanical stability. Invalid holes beyond the plate (mean: 145.51 ± 2.81 N) reduced structural strength more than those located under the plate (mean: 169.37 ± 3.34 N, *p* < 0.0001).

This finding suggests that bone drilling outside the plate coverage area may increase the risk of fixation failure. These findings provide valuable biomechanical insights for optimizing surgical techniques in clavicle hook plate fixation.

Numerous studies on femoral and tibial fracture fixation have demonstrated that empty screw holes act as stress concentrators, weakening mechanical stability (22, 23). Our findings further reveal that distal empty screw holes have a more significant impact on fixation strength than proximal empty holes. Preserving a proximal empty screw hole can reduce concentrated shear stress at the proximal clavicle, which may decrease the incidence of midshaft clavicle fractures. This observation suggests that adjusting the location of empty screw holes can help prevent postoperative fractures. There is limited research on how invalid holes in the clavicle affect fixation strength. However, previous studies have shown that cortical bone drilling reduces mechanical resistance, particularly when holes are located outside load-bearing areas (24). Our study found that invalid holes beyond the plate weakened structural strength more than those under the plate. This suggests that drilling outside the plate coverage area should be avoided to minimize the risk of fixation failure. Additionally, if an invalid hole is inadvertently drilled, using a longer plate to ensure the hole remains under the plate may help maintain stability.

This study makes several key academic contributions to evaluate multiple biomechanical factors. Unlike previous studies that focus on a single parameter, this study systematically analyzed plate bending, screw type, empty hole location, and invalid hole presence, providing a holistic understanding of clavicle hook plate fixation biomechanics. Identification of optimal fixation strategies. Our results suggest that forward bending, common screws, avoiding distal empty screw holes, and minimizing bone drilling beyond the plate coverage area significantly improve mechanical stability. These findings provide evidence-based recommendations for orthopedic surgeons. Bridging the knowledge gap in clavicle biomechanics. Most biomechanical studies focus on long bone fracture fixation, whereas clavicle fixation has unique biomechanical challenges (25–27).

Our study addresses this gap in the literature and may contribute to future improvements in clavicle implant design. These findings provide valuable new insights into clavicle fracture fixation mechanics and have direct clinical implications for improving surgical success rates and reducing implant failure risks. However, clavicular hook plates are primarily indicated for distal clavicle fractures (Neer type V) and acromioclavicular joint dislocations.

The forward-bent plate is unlikely to increase soft-tissue irritation or implant prominence because the degree of anterior curvature is mild and the distal clavicle region has substantial soft-tissue coverage. In contrast, the traditional plate is positioned more horizontally, often leaving a gap between its proximal end and the clavicular surface; under loading, the proximal screws may therefore be more prone to loosening or pull-out. With forward bending, the proximal end of the plate better conforms to the clavicular surface, which may reduce proximal screw loosening and pull-out under stress.

The forward-curved design is also unlikely to complicate implant removal. In our technique, removal is initiated by loosening the distal end, then applying gentle anteroposterior wobbling to gradually release the distal fixation. After the distal hook is mobilized, the proximal end is lifted while maintaining controlled wobbling to allow smooth, progressive extraction of the plate.

Despite its strengths, this study has some limitations that should be addressed in future research. This study used synthetic clavicle models for experimental consistency, but they do not fully replicate real human bone properties, such as cortical thickness, bone density, and fracture healing potential. The model does not include the acromioclavicular (AC) joint articulation or ligamentous structures; therefore, it cannot assess joint-constrained stability or clinically relevant instability patterns. Future studies should consider cadaveric or *in vivo* models for more clinically relevant results. This study primarily assessed axial loading until fracture, whereas physiological loads on the clavicle involve complex multidirectional forces (e.g., torsion, bending, and shear). Future studies should investigate multi-axial loading conditions to better simulate real-world mechanical stress. While we assessed initial fracture resistance, real-world implants experience cyclic loading over time, which may lead to fatigue failure. Future studies should explore fatigue testing and long-term implant stability.

Our experimental model simulates a Rockwood type III acromioclavicular joint dislocation. In clavicle hook plate fixation, we investigated risk factors for proximal-end stress fractures and strategies to prevent midshaft clavicular stress fractures. Based on our experimental results, modifications in surgical technique—such as plate bending, screw selection, and occluding unused holes—may reduce the incidence of stress fractures, offering practical guidance for clinical application.

This study provides biomechanical evidence, but clinical validation is necessary to confirm the impact of these factors on patient outcomes. Prospective clinical trials comparing different fixation strategies would help translate these findings into evidence-based surgical guidelines.

## Conclusion

This study systematically evaluated the biomechanical effects of plate bending, screw type, empty hole location, and invalid hole placement on clavicle hook plate fixation strength. The findings indicate that forward bending enhances mechanical stability, common screws provide superior fixation compared to locked screws, distal empty screw holes weaken structural integrity more than proximal ones, and invalid holes beyond the plate significantly reduce fixation strength. The findings of this study are based on a synthetic bone model; clinical translation will require further validation in cadaveric clavicle experiments. Future research should focus on cadaveric clavicle validation, multi-axial loading conditions, and long-term fatigue analysis to further refine these findings and enhance surgical decision-making.

## Data Availability

The original contributions presented in the study are included in the article/supplementary material, further inquiries can be directed to the corresponding authors.
